# Supplementary material for: Extreme Li-Mg selectivity via precise ion size differentiation of polyamide membrane
Source: Nat Commun. 2024 Mar 20;15:2505. doi: 10.1038/s41467-024-46887-4 (PMC10954764; doi:10.1038/s41467-024-46887-4)
Supplement: Supplementary file 1 — Supplementary Information [file 41467_2024_46887_MOESM1_ESM.pdf]

Supplementary Information

Extreme Li-Mg Selectivity via Precise Ion Size Differentiation of Polyamide Membrane

by Quan Peng, et al.

Table of contents

Supplemental methods

Supplemental Figures 1-16

Supplemental Tables 1-12

Contents

1. Supplementary Methods 1
1.1 Chemicals and Materials 1
1.2 Characterization 1
1.2.1 Scanning electron microscopy (SEM) 1
1.2.2 X-ray photoelectron spectroscopy (XPS) 1
1.2.3 Transmission electron microscopy (TEM) 2
1.2.4 Total organic carbon (TOC) 2
1.2.5 Streaming potential measurement 2
1.2.6 Interfacial tension measurement 2
1.3 Determination of MWCO and pore size distribution of PA NF membranes 2
1.4 SDEM model 3
2. Supplementary figures 4
2.1 Variation of water/hexane interfacial tension and PIP concentration near the interface with the change of DDP concentration in hexane 4
2.2 Surface chemical composition and zeta potential of the PA NF membranes prepared from traditional IP and OSARIP 6
2.3 Pore structure and Desalination performance of the PA NF membranes prepared from OSARIP 9
2.4 $MgCl_2$ rejection of NF membranes reported in literatures 13
2.5 Size, hydration energy, and corresponding rejection of various cations 14
2.6 MWCO, pore size distribution, and desalination performance of DK membrane 16
2.7 Performance of the PA NF membrane prepared from OSARIP for separating Li^+ and Mg^{2+} from simulated brine 17
2.8 Summary of Li/Mg selectivity of various membranes 20
2.9 PA NF membranes prepared from different conditions 23
2.10 PA NF membranes prepared from OSARIP by using PEI and TMC as the monomers 24
3. Supplementary references 26

1. Supplementary Methods

1.1 Chemicals and Materials

Trimesoyl choride (TMC, 98%) was purchased from Sigma-Aldrich and piperazine (PIP, 99%) was purchased from Aladdin. Didecyl phosphate (95%) was purchased from TCI and monododecyl phosphate (90%) was purchased from Alfa. Commercial polyethersulfone (PES) ultrafiltration membrane with MWCO of 50000 Da was purchased from Risingsun Membrane Technology Co., Ltd. (Beijing, China). Na_2SO_4 (≥99%), $MgSO_4$ (≥99%), $MgCl_2·6H_2O$ (≥99%), $CaCl_2·2H_2O$ (≥99%), NaCl (≥99.5%), LiCl (99%), $ZnCl_2$ (99%), $CuCl_2$ (99%), $NiCl_2$ (99%), $CoCl_2$ (99%), $BaCl_2$ (99%), $CuCl_2$ (99%), KCl (99%), RbCl (99%), CsCl (99%), glycerol (99%), xylose (99%), sucrose (99%), glucose (99%), n-hexane (97%) and raffinose (98%) were all brought from Innochem (Beijing, China).

1.2 Characterization

1.2.1 Scanning electron microscopy (SEM)

SEM images were characterized by a Su8200 (Hitachi, Japan) field emission scanning electron microscopes (FE-SEM). Before characterization, the membrane samples were dried in an oven at 60 ºC for 30 minutes. Subsequently, they were sprayed with platinum at 20 mA for 1 minute.

1.2.2 X-ray photoelectron spectroscopy (XPS)

The surface chemical compositions of PA NF membranes were analyzed using a X-Ray photoelectron spectroscopy (Thermo Fisher Scientific ESCALAB Xi+). For XPS measurement, high-resolution scans were conducted for carbon, nitrogen, and oxygen regions. The resulting XPS spectra were analyzed using a XPSPEAK41 software. The crosslinking degree of PA selective layers were determined based on the atomic content ratio of O and N elements detected by XPS.[1,2]

1.2.3 Transmission electron microscopy (TEM)

Cross-sectional TEM images of PA NF membranes were obtained on a Tecnai G2 F20 S-TWIN field emission TEM. To prepare TEM samples, the PA NF membranes were first cut into 2 mm wide strips using a microtome (Leica EM UC7). Then the samples were embedded in epoxy resins and dried at 60°C for 24 hours before placed on carbon support grids.

1.2.4 Total organic carbon (TOC)

The concentration of neutral molecules in the feed solution and permeate were measured using the OI Analytical Aurora Model 1030 through total organic carbon (TOC) analysis.

1.2.5 Streaming potential measurement

The surface zeta potentials of PA NF membranes were measured on an electro-kinetic analyzer (SurPASS3, Anton Paar) with a pH scanning range of 2 to 10.5.

1.2.6 Interfacial tension measurement

An interfacial tensiometer (BZY200, Shanghai Fangbo) was used to measure the interfacial tension between PIP aqueous solution and TMC hexane solution containing DDP in different concentrations.

1.3 Determination of MWCO and pore size distribution of PA NF membranes

The MWCO of the PA NF membranes was determined by a standard method of measuring the rejection of neutral molecules, such as glycerol (92 Da), xylose (120 Da), glucose (180 Da), sucrose (342 Da), and raffinose (504 Da), by the membranes. The aqueous solution of these neutral molecules with a concentration of 200 ppm was used as feed to pass through the membrane at 4 bar. The rejection of neutral molecules was calculated according to the concentration of the feed and permeate qualified by TOC. The MWCO of the membranes was equal to the molecular weight of the neutral molecule with a rejection of 90%.

With the premise of no steric and hydrodynamic interactions between these neutral solutes and the pores of the membranes, the corresponding pore size distribution can be expressed by the probability density function (PDF) (Eq. (1)).

$$\frac{\mathrm{d}R(d_p)}{\mathrm{d}d_p}=\frac{1}{d_p\ln\sigma_p\sqrt{2\pi}}\exp\left[-\frac{(\ln d_p-\ln\mu_p)^2}{2(\ln\sigma_p)^2}\right] \quad (1)$$

where d_p is the Stokes diameter of the neutral molecules, μ_p is the mean pore size and σ_p is the geometric standard deviation of the PDF curve. The μ_p is equals to the d_p of the neutral solute with a rejection of 50%. σ_p represents the distribution of the membrane pore size, which is the ratio of d_p of the neutral molecule with a rejection of 84.13% to that of 50%. The d_p of the neutral solutes is calculated according to Eq. (2).

$$\lg\frac{d_p}{2}=-1.4962+0.4654\lg\mathrm{M} \quad (2)$$

The pore size range of PA NF membranes is determined from the PDF curve using the data extraction tool (Getdata), where all pores with probability density greater than 0.01 in the curve are included to determine the distribution range of pore size from minimum to maximum.

1.4 SDEM model

The mass transport of mixed electrolyte solutions across the PA NF membrane can be described by the solution-diffusion-electromigration (SDEM) model.[3-5] The ion flux for species i, J_i, in the SDEM model is described using the modified Nernst-Planck Eq. (3):

$$J_i=-P_i\left(\frac{\mathrm{d}c_i}{\mathrm{d}x}+z_ic_i\frac{\mathrm{d}\varphi}{\mathrm{d}x}\right) \quad (3)$$

where P_i is the ion permeability, c_i is the ion concentration in a virtual solution that is in thermodynamic equilibrium with the membrane phase, x is the transmembrane coordinate normalized by the membrane thickness, z_i is the valence of species i, and φ is the dimensionless electrical potential in the virtual solution. The ion flux and ion rejections can be determined by solving Eq. (3) with charge neutrality (Eq. 4) and steady-state conditions (Eq. 5):

$$\sum_i z_i c_i = 0 \quad (4)$$

$$J_i = J_\mathrm{w} c_{\mathrm{p},i} \quad (5)$$

where J_w is the permeate flux, and $c_{\mathrm{p},i}$ is permeate concentration of ion i. J_w can be measured experimentally or estimated by equation 6:

$$J_\mathrm{w} = P_\mathrm{w}(\Delta P - \Delta\pi) \quad (6)$$

where P_w is the water permeability, ΔP and $\Delta\pi$ are hydrostatic pressure difference and osmotic pressure difference across the membrane, respectively.

Thus, the selective ion transport between Li^+ and Mg^{2+} across the NF membrane is characterized by the difference of ion permeability in the SDEM model. Ion permeability, P_i, can be fitted from experimental rejection results, given the used feed composition and measured permeate flux. The fitting applies least squares method, i.e., minimizing the sum of the squares of the residuals between experimentally measured ion rejections and rejections predicted by the SDEM model. More details about the SDEM model and its application to describe the selective separation of Li^+ and Mg^{2+} can be found elsewhere.[5]

2. Supplementary figures

2.1 Variation of water/hexane interfacial tension and PIP concentration near the interface with the change of DDP concentration in hexane

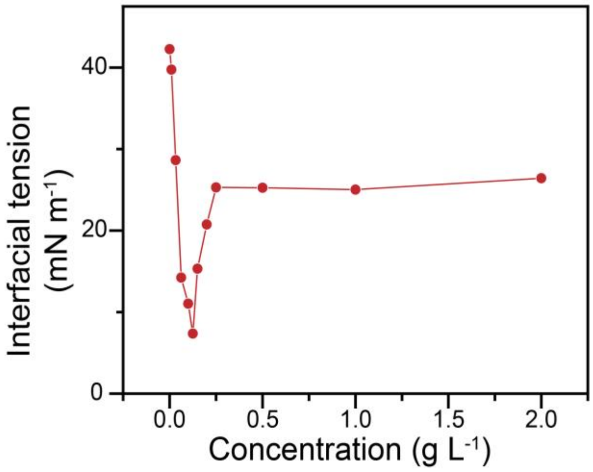

Supplementary Fig. 1. Variation of the interfacial tension of water and hexane as a

function of DDP concentration in hexane.

Note: The critical concentration of DDP in hexane can be determined through the utilization of an interfacial tensiometer to monitor the variation of oil/water interfacial tension with respect to DDP concentration in hexane. As demonstrated in Supplementary Fig. 1, the interfacial tension rapidly decreases and reaches a minimum at a concentration of 0.1 g L^{-1}. Increasing the concentration of DDP in hexane further results in an increase in interfacial tension, reaching a value of 27 mN m^{-1} at around 0.25 g L^{-1}, after which no further change is observed. The change of water/hexane interfacial tension may attribute to the transition in the molecular arrangement structure of the DDP assembly.

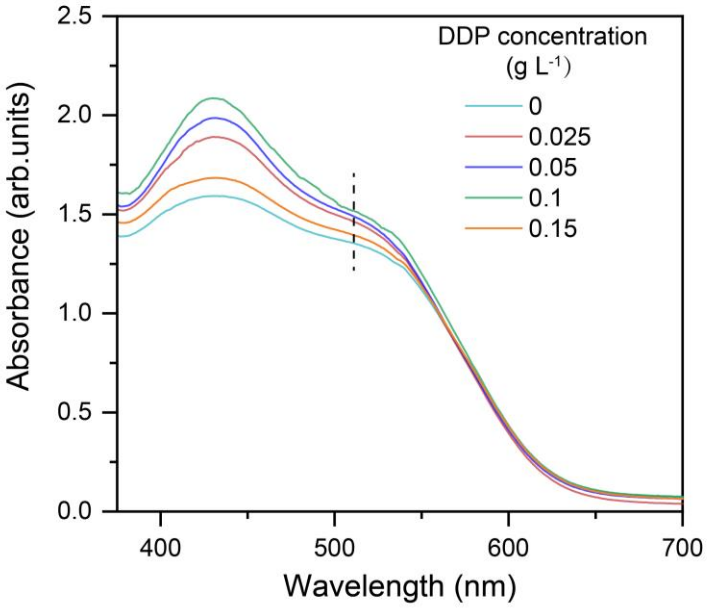

Supplementary Fig. 2. UV-vis absorption spectra of PIP aqueous solution near the water/hexane interface with different concentration of DDP in hexane.

Note: In order to confirm the effect of the DDP assembly on accumulating the PIP molecules at the interface and facilitating the PIP diffusion to across the interface, we conducted a UV-vis absorption measurement to examine the PIP concentration near the interface following the method developed by Zhu et al.[6] In the UV-vis absorption spectra, the absorbance peak of PIP is at 512 nm. Upon increasing the concentration of DDP in the hexane solution, the UV-vis absorbance correspondingly increases and reaches its maximum level at a concentration of 0.1 g L^{-1}, which is consistent with the

variation of interfacial tension to the change of DDP concentration shown in Supplementary Fig. 1.

2.2 Surface chemical composition and zeta potential of the PA NF membranes prepared from traditional IP and OSARIP

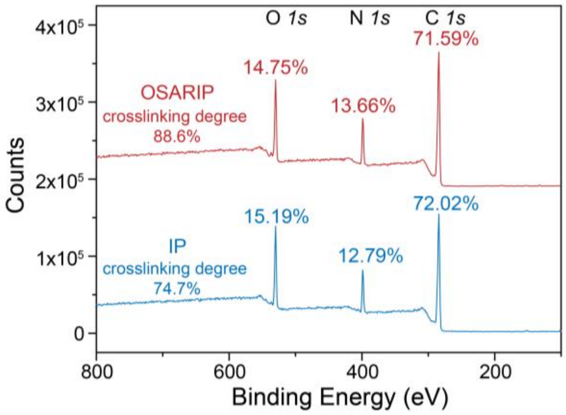

Supplemental Fig. 3. Surface chemical elemental composition of PA membranes prepared from OSARIP (red line) and traditional IP (blue line) and the crosslinking degree of the membrane calculated from the atomic ratio of O/N.

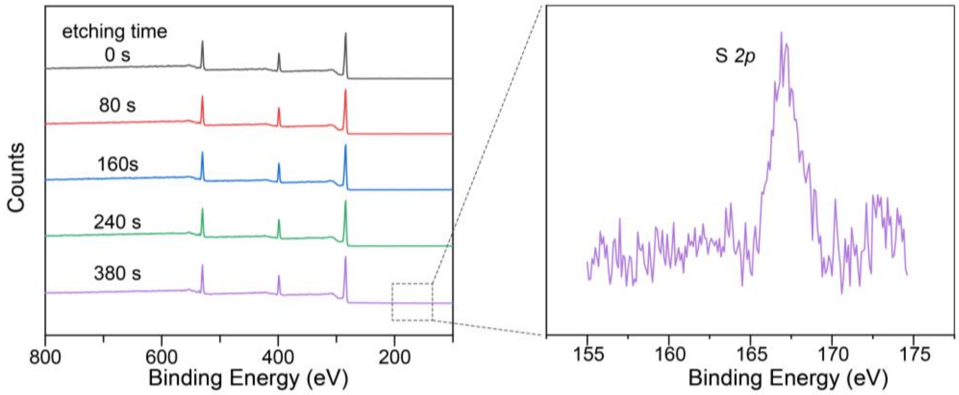

Supplemental Fig. 4. XPS survey spectra of PA membrane prepared from OSARIP with different etching time.

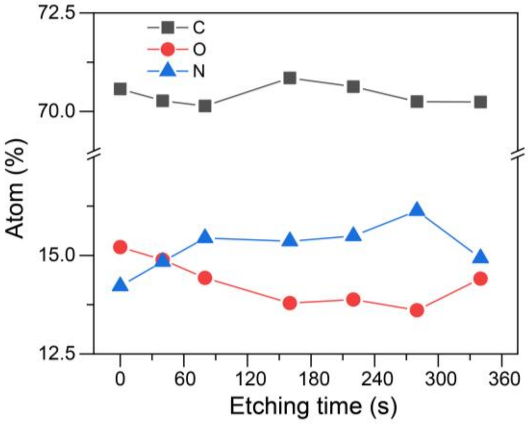

Supplementary Fig. 5. In-depth analysis of C, N, and O content in the PA active layer using the combination of XPS measurement and Ar ion sputtering technique.

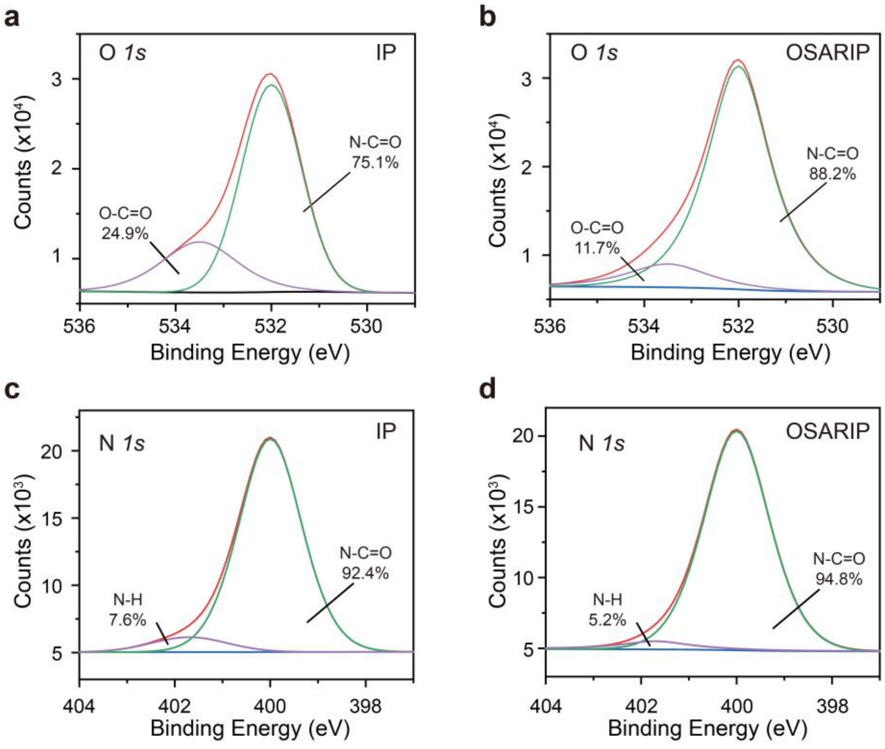

Supplementary Fig. 6. O1s and N1s XPS spectra of the PA membranes prepared from traditional IP and OSARIP. (a, b) O 1s spectra and (c, d) N 1s spectra.

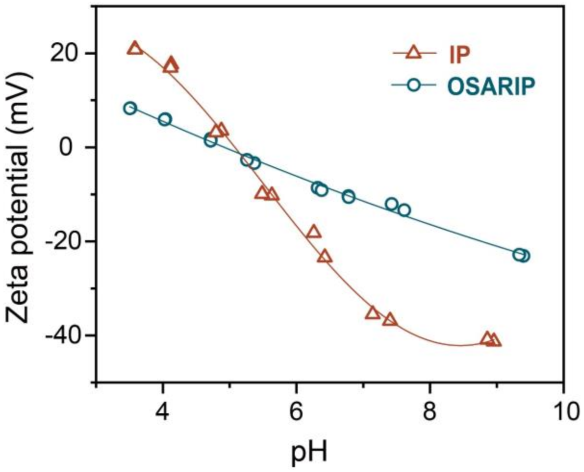

Supplementary Fig. 7. Surface zeta potential of PA NF membranes prepared from traditional IP and OSARIP.

Note: In order to detect the surface crosslinking degree of the PA NF membranes prepared from traditional IP and OSARIP, we used the XPS technique to detect the surface chemical element composition. The XPS survey spectra of the membrane prepared from OSARIP and traditional IP is shown in Supplemental Fig. 3. According to the atomic ratio of O/N detected by XPS, we calculated that the surface crosslinking of the membrane prepared from OSARIP and traditional IP, which is 74.7 % and 88.6% respectively. With the Ar ion sputtering technique, we further performed an in-depth analysis of the chemical elemental composition of the PA NF membrane prepared from OSARIP by XPS. As depicted in Supplementary Figs. 4 and 5, the N atomic content exhibits a gradual increase with increasing etching time, while the O content shows a corresponding decrease. This trend suggests that the inner space of the PA active layer possesses a higher concentration of amine groups compared to the surface. Notably, the O/N atomic ratio within the inner space is less than 1, significantly lower than the surface, indicating a predominance of amine-terminated chemical structures. Prolonging the etching time to 360 seconds leads to the detection of a small amounts of sulfur (S), attributed to the underlying PES support. At this depth, the N content slightly diminishes, while the O content rebounds. These collective observations point towards a heterogeneous structure within the PA layer along the depth axis. Based on the change in the O/N atomic ratio with respect to etching time, the inner part of the membrane appears to be denser than the front and back surfaces.

The surface chemical groups of the PA NF membranes were revealed by fitting the O *1s* and N *1s* peaks of XPS survey spectra according to the binding energy of various chemical functional groups, as shown in Supplementary Fig. 6. It shows that the content of carboxyl group (O-C=O) and amine group (N-H) in the PA NF membrane prepared from OSARIP is less than that of the PA NF membrane prepared from traditional IP. This indicates that the reaction degree between PIP and TMC in the PA NF membrane prepared from OSARIP is greater than in the PA NF membrane prepared from traditional IP. Because the surface charge of the PA NF membrane is primarily determined by the amount of amine and carboxyl groups on its surface, the decrease in the content of these groups enables the surface of the membrane less charged, as evidenced by the change of surface zeta potential of the membranes with respect to the pH (Supplementary Figure 7).

2.3 Pore structure and Desalination performance of the PA NF membranes prepared from OSARIP

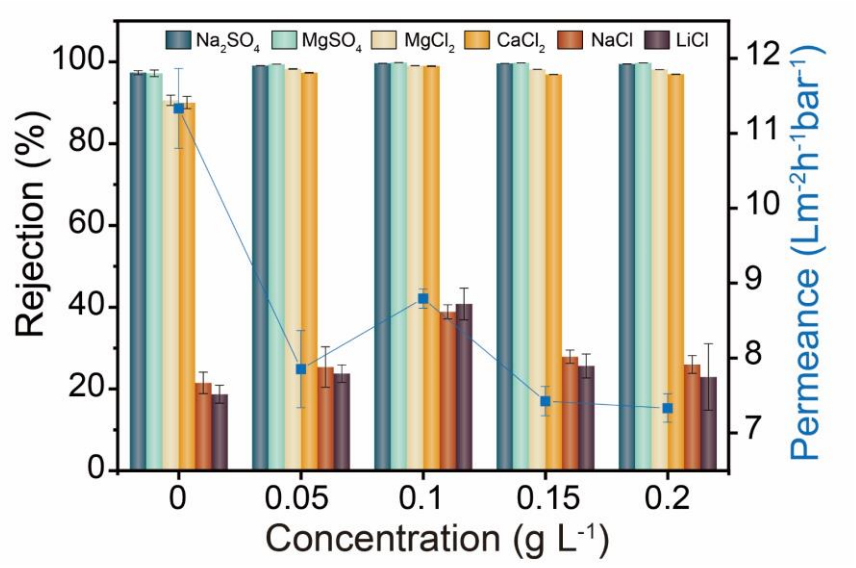

Supplementary Fig. 8. Desalination performance of the PA NF membranes prepared from OSARIP with different concentration of DDP in hexane. Error bar represents the standard deviation of three replicate measurements.

Supplementary Table 1. Data list of salt rejection and water permeance of the PA NF membranes prepared from OSARIP with different concentration of DDP in hexane. Error bar represents the standard deviation of three replicate measurements.

C_{DDP}	R_{salt} (%)						Pw
(g L^{-1})	Na_2SO_4	$MgSO_4$	$MgCl_2$	$CaCl_2$	NaCl	LiCl	($Lm^{-2}h^{-1}bar^{-1}$)
0	97.37±0.48	97.23±0.79	91.21±1.26	90.87±1.48	23.34±2.62	20.26±2.19	11.33±0.53
0.05	99.08±0.02	99.44±0.01	98.28±0.05	97.37±0.13	25.32±4.9	23.74±2.14	7.85±0.51
0.10	99.66±0.01	99.84±0.01	99.08±0.04	99.00±0.09	38.86±1.73	40.78±3.88	8.79±0.13
0.15	99.63±0.01	99.75±0.01	98.18±0.02	96.95±0.04	27.89±1.62	25.63±2.92	7.425±0.19
0.20	99.48±0.03	99.75±0.01	98.14±0.02	97.00±0.05	25.99±2.17	22.40±8.13	7.33±0.05

C_{DDP}: DDP concentration in hexane; R_{salt}: salt rejection; P_w: water permeance.

Note: The salt rejection and water permeance of PA NF membranes prepared from OSARIP with different concentrations of DDP in hexane are presented in Supplementary Fig. 5 and Table 1. Increasing the concentration of DDP in hexane can effectively enhance the rejection of divalent salts, such as Na_2SO_4, $MgSO_4$, $MgCl_2$, and $CaCl_2$. At the concentration of 0.1 g L^{-1}, the resulting membrane exhibits the highest rejection to the salts of Na_2SO_4, $MgSO_4$, $MgCl_2$, and $CaCl_2$, all of which are higher than 99%. Meanwhile, the rejections of monovalent salts, including NaCl and LiCl, are around 20-40%. Further increasing the concentration of DDP leads to a slight decrease in salt rejection. The salt rejection as a function of DDP concentration indicates that 0.1 g L^{-1} DDP is the optimal condition for preparing PA NF membrane with the best desalination performance. In the following text, all PA NF membranes were prepared at 0.1 g L^{-1} DDP unless stated otherwise.

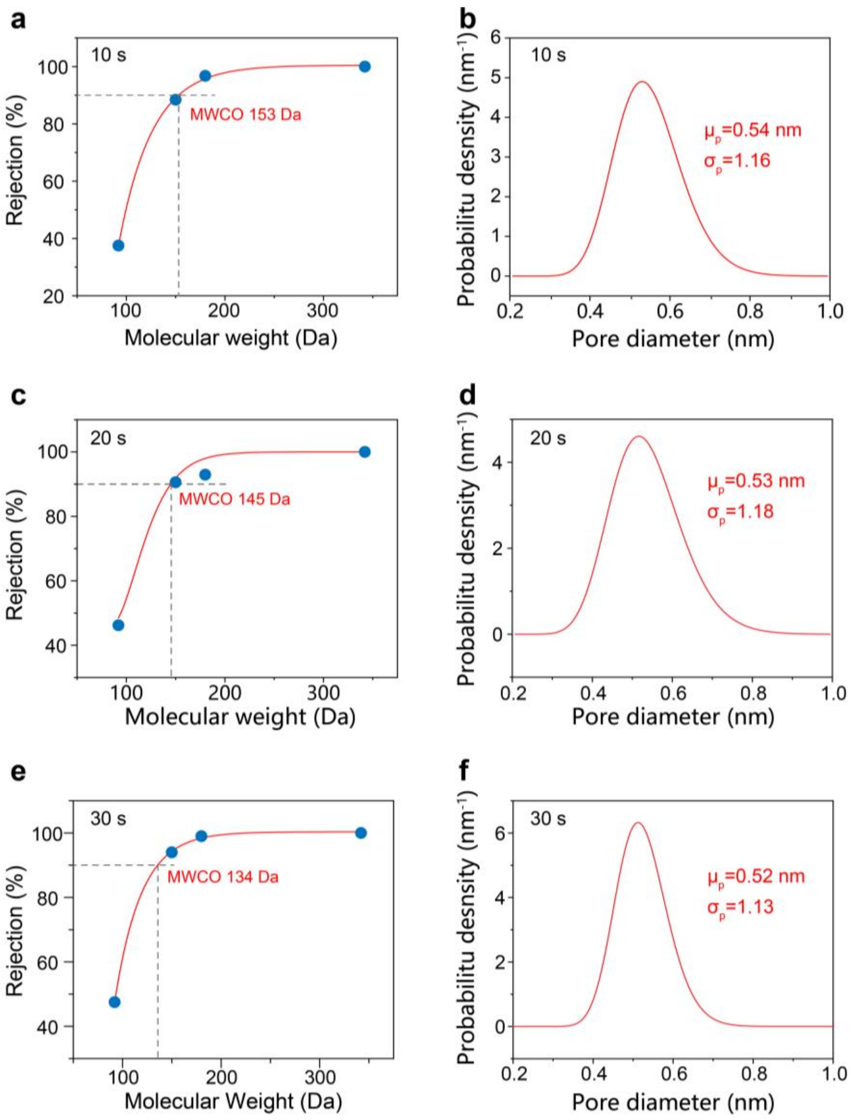

Supplementary Fig. 9. MWCO and pore size distribution of PA NF membranes prepared from OSARIP with the reaction time of (a, b) 10 s, (c, d) 20 s, and (e, f) 30 s.

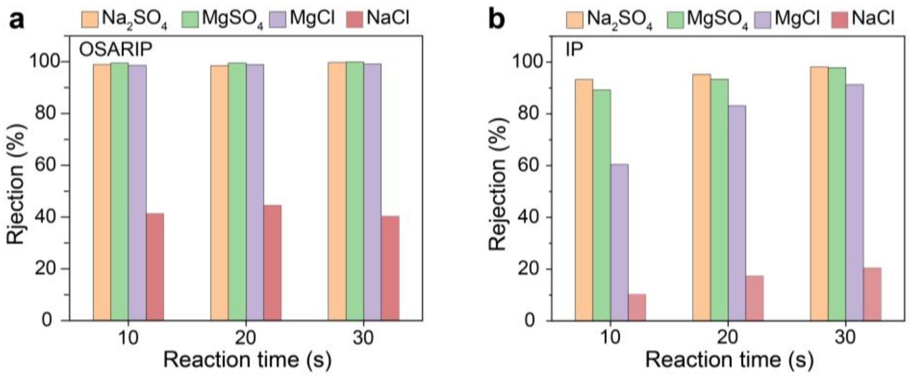

Supplementary Fig.10. Salt rejection of PA membranes prepared from (a) OSARIP and (b) traditional IP with different reaction time.

Note: To evaluate the effect of surfactant assembly on the IP kinetic, we investigate the reaction time on the structure and properties of the PA NF membrane prepared via OSARIP. The reaction time was changed from 10 s to 30 s. To assess the relationship between reaction time and membrane characteristics, we analyzed the pore size distribution of the PA membrane using the rejection of charge-neutral molecules, and the desalination performance of the resulting PA NF membranes. Supplementary Figs. 9, 10 present the obtained results.

The surfactant-assembly can significantly accelerate the IP reaction, enabling the PA membrane prepared from OSARIP with a 10 s reaction time to exhibit a remarkably low molecular weight cut-off (MWCO) of 154 Da (Supplementary Fig. 9a). This value is considerably smaller compared to the PA membrane prepared from traditional IP with a 30 s reaction time. Furthermore, the pore size distribution of the surfactant-assisted membrane was notably narrower (Supplementary Fig. 9b). Increasing the reaction time to 20 and 30 s yielded PA NF membranes with MWCO of 145 Da and 134 Da, respectively, suggesting that prolonged the reaction did not significantly affect the pore structure of the membrane prepared from OSARIP (Supplementary Figs. 9c-9f) .

Consistent with the minimal pore size variation, the three OSARIP-derived PA NF membranes exhibited comparable salt rejection performance (Supplementary Fig. 10a). Notably, they achieved very high rejection (around 99%) for all divalent salts tested, including Na_2SO_4, $MgSO_4$, and $MgCl_2$. In contrast, the PA membrane prepared from traditional IP displayed a gradual increase in salt rejection with increasing reaction time. For instance, $MgCl_2$ rejection rose from 60.4% to 91.3% as the reaction time extended from 10 to 30 s (Supplementary Fig. 10b). This minimal deviation in pore size and salt rejection across different reaction times for the membranes prepared from OSARIP indicates a rapid transition to a self-limited polymerization state. This observation further proves the promoting effect of oil-soluble surfactant assembly at the oil/water interface on the IP kinetics.

2.4 $MgCl_2$ rejection of NF membranes reported in literatures

Supplementary Table 2. List of the $MgCl_2$ rejection and the corresponding MWCO of the NF membranes reported in the literatures.

Membrane	MWCO (Da)	$MgCl_2$ rejection (%)	Ref.
**OSARIP**	134	99.13	This work
**IP**	258	90.61	This work
**PIP-TMC**	335	46.0	9
**SDS**	245	94.5	9
**STS**	212	98.2	9
**SHS**	134	98.1	9
**SDS**	208	95.0	10
**SDBS**	224	82.0	10
**SB3-14**	220	89.6	10
**CTAB**	302	66.0	10
**PIP-TMC**	274	45.5	10
**TsNA**[#]	255	50.0	10
**BTC–PIP**	240	99.1	11
**TFC-2-1PIP**	285	73.0	12
**TFC-0-1PIP**	326	90.0	12
**PES-CNT-PA**	353	93.2	13
**PA20**	360	72.2	14
**CSP0**	320	78.2	15
**CSP6**	250	71.0	15
**PIP/TMC**	200	88.5	15
**PIP/TMC/SWCNT**	397	64.9	16
**TFN-50**	308	45.0	17
**H-TFC NF**	170	94.0	18
**PDA/PEI**	190	95.8	19
**TMC/TAEA**	388	81.0	20
**TMC/PIP**	350	83.0	20
**PIP/β-CD/TMC**	530	20.0	21
**TMC@-15**	430	42.6	22
**TMC@5**	400	50.8	22
**TMC@20**	385	61.0	22
**TMC@35**	375	70.5	22
**TMC@50**	360	86.0	22
**PA@A-0**	353	38.0	23
**PA@20-3**	316	46.0	23
**PA@50-3**	300	63.0	23
**PA@80-3**	273	67.0	23
**2D BFS/TMC/PIP**	345	35.0	24

**PA@W-14**	306	33.0	25
**PA@W-0**	300	78.0	25
**PA@W-7**	245	90.0	25
**PEI-g-PA**	300	35.5	26
**THPC-5**	348	34.0	27
**PA/M-50**	384	11.4	28
**MBA50**	320	32.0	29
**BDSA/PIP**	400	45.5	30
**HL**	300	82.9	31
**PIP/TMC/TMA**	546	32.0	32
**NF40**	180	99.0	33
**UTC20**	180	92.0	33
**UTC60**	180	83.6	34
**NF90**	180	92.0	35
**NF270**	300	55.0	35
**Desal 5 DK**	300	75	36

Note: All the membranes are negatively charged.

2.5 Size, hydration energy, and corresponding rejection of various cations

Supplementary Table 3. The size and hydration energy of the cations and their corresponding rejections by the PA NF membrane prepared from OSARIP.[7,8]

	Ionic Radius (Å)	Stokes Radius (Å)	Hydration Radius (Å)	Hydration Energy (kcal mol^{-1})	Salt	R (%)
Zn^{2+}	0.74	3.49	4.3	467.3	$ZnCl_2$	99.21%
Co^{2+}	0.72	3.35	4.23	457.7	$CoCl_2$	99.04%
Mg^{2+}	0.65	3.47	4.23	437.4	$MgCl_2$	99.13%
Cu^{2+}	0.72	3.25	4.19	480.4	$CuCl_2$	99.15%
Ca^{2+}	0.99	3.1	4.12	359.7	$CaCl_2$	99.03%
Ni^{2+}	0.7	2.92	4.04	473.2	$NiCl_2$	99.01%
Ba^{2+}	1.35	2.9	4.04	298.8	$BaCl_2$	98.36%
Li^{+}	0.6	2.38	3.82	113.5	LiCl	40.78%
Na^{+}	0.95	1.84	3.58	87.2	NaCl	38.86%
K^{+}	1.33	1.25	3.31	70.5	KCl	36.46%
Rb^{+}	1.48	1.18	3.29	65.7	RbCl	41.27%
Cs^{+}	1.69	1.19	3.29	59.8	CsCl	42.33%

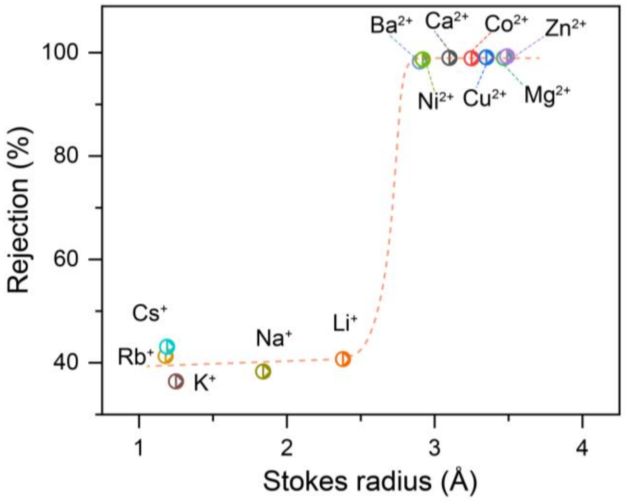

Supplementary Fig. 11. The rejection of cations by PA NF membranes prepared from OSARIP as a function of Stokes radius. Error bar represents the standard deviation of three replicate measurements.

Note: The selectivity of the PA NF membrane prepared from OSARIP for separating monovalent and divalent cations was evaluated by passing the chloride salt of the cation aqueous solution with a concentration of 1000 ppm through the membrane. The rejection rates of these cations are presented in Supplementary Fig. 11 and Supplementary Table 3. The membrane displayed high rejection rates of up to 99% to all divalent cations, while showed a relatively low rejection rates to all monovalent cations. This suggests the outstanding ability of the PA NF membrane for separating divalent and monovalent cations. The surface of the PA NF membrane prepared from OSARIP was found to be negative from the result of surface streaming potential measurement as shown in Supplementary Fig. 4. Hence, the rejection of the membrane to all cations is primarily induced by the size-sieving effect, rather than Donnan exclusion. Upon correlating the rejection to the Stokes radius of the cations, a sharp transition region between the size range of 0.6 Å was observed, indicating the ultra-precise size separation capability of the PA NF membrane.

2.6 MWCO, pore size distribution, and desalination performance of DK membrane

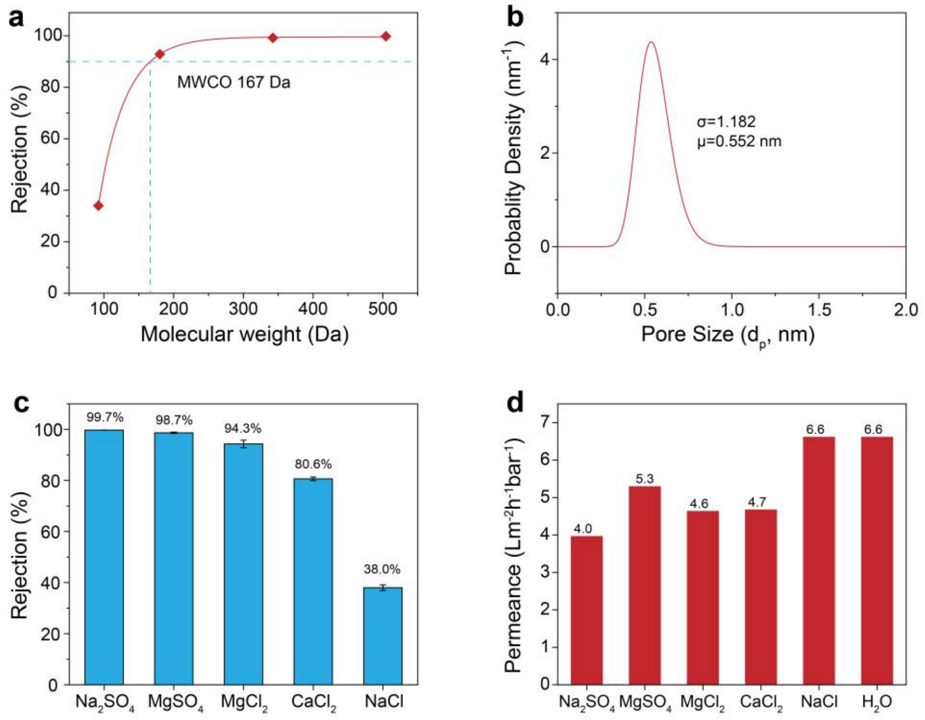

Supplementary Fig. 12. (a) Rejection of neutral molecules, including glycerol, glucose, sucrose, and raffinose, by the commercial DK membrane. (b) Pore size distribution of the DK membrane derived from the rejection curve of neutral molecules. (c) Salt rejection and (d) corresponding permeance of the DK membrane using single salt aqueous solution as feed. Error bar represents the standard deviation of three replicate measurements.

Note: Utilizing neutral molecules with different molecular weights as probes, we analyzed the pore size of the commercial DK membrane. The membrane displayed a MWCO of 166 Da, which is smaller than most of NF membranes. The rejection curve of neutral molecules indicated a pore size distribution parameter of 1.182, which suggests a relatively narrow pore size distribution of the membrane. The membrane showed a high rejection to divalent salts with 99.7% of Na_2SO_4, 98.7% of $MgSO_4$, and 94.3% of $MgCl_2$. The corresponding permeance of these salts and pure water are shown in Supplementary Fig. 12.

2.7 Performance of the PA NF membrane prepared from OSARIP for separating Li^+ and Mg^{2+} from simulated brine

Supplementary Table 4. The rejection of Li^+ and Mg^{2+} and Li^+/Mg^{2+} selectivity as a function of Mg^{2+}/Li^+ mass ratios of feed solution. The salt concentration of the binary salt mixture is 2000 ppm. Error bar represents the standard deviation of three replicate measurements.

Mg^{2+}/Li^+ mass ratios	feed (ppm)		permeate (ppm)		Li^+ Rejection (%)	Mg^{2+} Rejection (%)	$S_{Mg/Li}$
	Li^+	Mg^{2+}	Li^+	Mg^{2+}			
10:1	44.4±2.1	542.6±24.3	52.06±2.72	1.16±0.27	-17.25	99.79	548
20:1	21.8±1.7	497.0±26.8	32.34±3.37	0.89±0.35	-48.34	99.82	828
40:1	12.8±1.4	542.7±21.3	20.51±2.91	0.51±0.27	-60.23	99.91	1705
60:1	8.4±0.9	535.6±28.1	12.36±2.39	0.19±0.07	-47.14	99.96	4147

Supplementary Table 5. The rejection of Li^+ and Mg^{2+} and the Li^+/Mg^{2+} selectivity as a function of the concentration of feed solution. The Mg^{2+}/Li^+ mass ratio of the binary salt mixture is 20:1. Error bar represents the standard deviation of three replicate measurements.

Concentration (ppm)	Feed (ppm)		Filtrate (ppm)		Li^+ Rejection (%)	Mg^{2+} Rejection (%)	$S_{Mg/Li}$
	Li^+	Mg^{2+}	Li^+	Mg^{2+}			
2000	21.8±1.7	497.0±26.8	32.34±3.3	0.9±0.3	-48.35	99.82	824
3000	29.5±2.1	720.7±34.1	45.1±4.3	5.3±1.2	-52.71	99.25	203
4000	42.4±4.7	1059.0±36.8	73.4±5.5	8.9±2.3	-73.21	99.16	206
5000	56.7±5.3	1393.5±35.6	110.2±6.3	14.6±3.1	-94.35	98.95	185

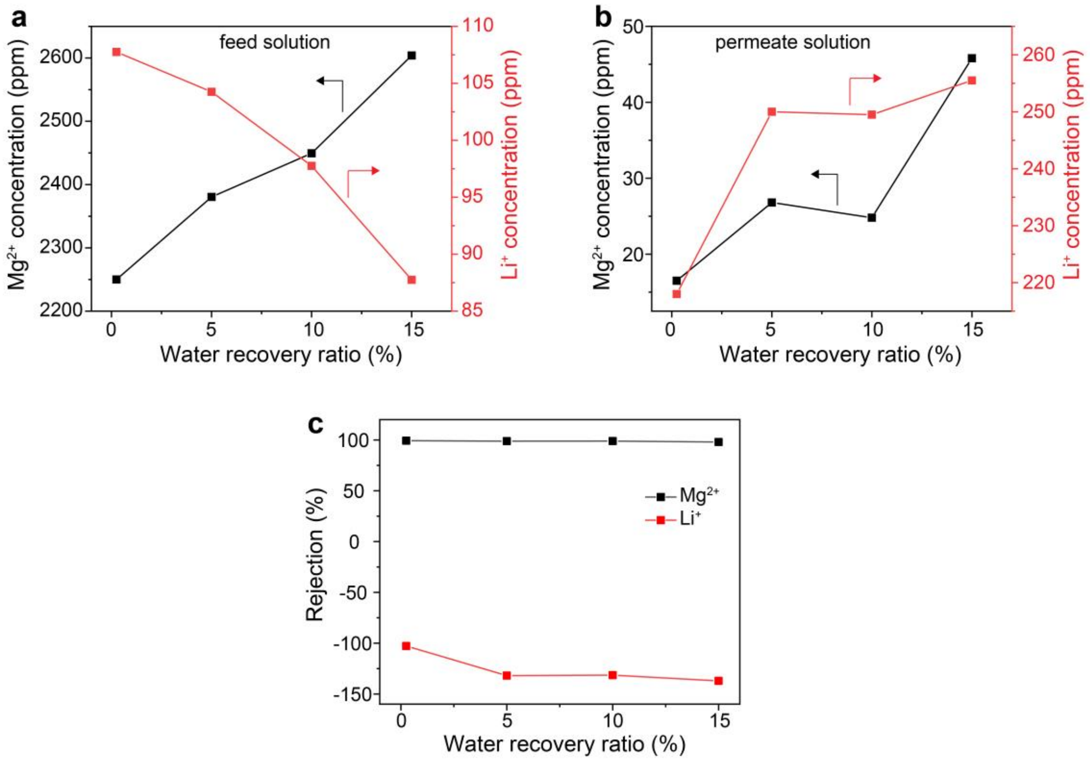

Supplementary Fig. 13. Variation of the concentration of Mg^{2+} and Li^{+} in the (a) feed and (b) permeate as a function of water recovery ratio. (c) The rejection of Mg^{2+} and Li^{+} by the PA NF membrane prepared from OSARIP as a function of water recovery ratio measured under cumulative model. The water recovery ratio is defined as the volume of collected permeate divided by the initial volume of the feed solution. Notably, the total salt concentration of the feed solution is about 10000 ppm and the Mg^{2+}/Li^{+} mass ratio of this feed solution is 20:1. The operation pressure is 6 bar.

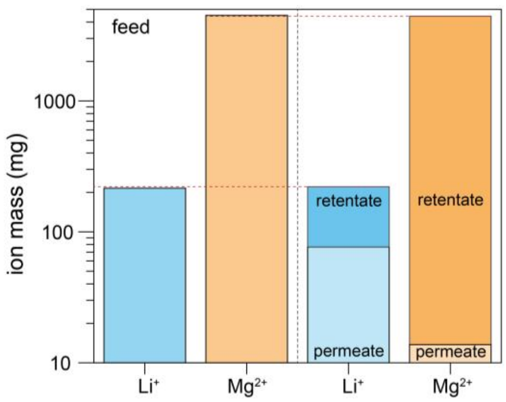

Supplementary Fig. 14. The mass of Li^{+} and Mg^{2+} in the feed, permeate and retentate. The water recovery ratio is 15%.

Supplementary Table 6. The ion composition of feed and permeate solutions.

Ion	Concentration (ppm)		Rejection (%)	Li^+/Mg^{2+} selectivity
	feed	permeate		
Mg^{2+}	2271	23	98.99	212
Li^+	107	229.5	-114.49	
Na^+	78.2	162.5	-107.67	
K^+	108	218.5	-102.31	
Ca^{2+}	21.2	undetected		

Note: The strong negative rejection of Li^+ during the separation can rapidly change feed solution concentration and permeate solution concentration. That means the water recovery may have a significant on the Li^+/Mg^{2+} selectivity. Therefore, we monitored the changes in feed and permeate concentrations, as well as Li^+ and Mg^{2+} rejections, with respect to permeate volume using a simulated brine with a concentration around 10000 ppm as feed. In this feed solution, the Mg^{2+}/Li^+ mass ratio is 20:1 and has no other metal ions. As shown in Supplementary Fig. 13a, it clearly demonstrates a decrease in feed Li^+ concentration with increasing water recovery ratio (defined as the volume of collected permeate divided by the initial volume of the feed solution), which is induced by the negative rejection of Li^+. Conversely, the high Mg^{2+} rejection leads to a rapid increase in its feed concentration, consequently raising the Mg^{2+}/Li^+ mass ratio. As previously shown in Fig. 3a in the manuscript, higher Mg^{2+}/Li^+ mass ratios lead to stronger negative Li^+ rejection. This explains the observed increase in Li^+ concentration in the permeate with increasing water recovery ratio (Supplementary Fig. 13b). Additionally, the rising Mg^{2+} feed concentration is prone to increase its permeation, leading to a higher Mg^{2+} concentration in the permeate as well. Due to the dynamic changes in Li^+ and Mg^{2+} concentration in both feed and permeate, the cumulative rejections of Li^+ changed from -102.8% to -137.1% with an increase in water recovery ratio from 0 to 15% (Supplementary Fig. 13c). At the same time, the cumulative rejection of Mg^{2+} changed from 99.3% to 98.8%. This suggests that a cumulative

Li^+/Mg^{2+} selectivity of 197 is obtained with a water recovery ratio of 15%. This selectivity is still higher than that achieved by the vast majority of other PA NF membranes, indicating the exceptional superiority of the PA NF membrane prepared from OSARIP for the separation of Li^+ and Mg^{2+}. According to the concentration of feed solution, permeate, and retentate, we performed a mass balance calculation by neglecting the minor influence of aliquots. The results are displayed in Supplementary Fig. 14. The total mass of Li^+ and Mg^{2+} in the permeate and retentate combined was essentially identical to the mass in the feed solution, with a minor discrepancy of around 1%. We attribute this minor difference to the aforementioned aliquots.

In practical industrial applications of NF technology for lithium extraction from brine, feed solutions typically exhibit higher concentrations and contain additional metal ions, such as Na^+, K^+, and Ca^{2+}. To simulate the feed solution used in practical application, we used a mixed salt solution with total salt concentration of up to 10000 ppm, in which other metal ions, such as Na^+, K^+, and Ca^{2+}, are also contained. Even under this condition, the PA NF membrane demonstrates exceptional Li^+/Mg^{2+} selectivity more than 200. This remarkable performance underscores the unrivaled superiority of membranes with uniform pore sizes for lithium extraction from salt lake brine.

2.8 Summary of Li/Mg selectivity of various membranes

Supplementary Table 7. Summary of Li^+/Mg^{2+} selectivity of the membranes prepared from OSARIP and previously reported membranes.

Membrane	Mg^{2+}/Li^+ mass ratio	Applied pressure	Feed concentration (ppm)	$S_{Mg/Li}$	Ref.
**OSARIP**	10:1	0.4 MPa	2000	548	This work
**OSARIP**	20:1	0.4 MPa	2000	828	This work
**OSARIP**	40:1	0.4 MPa	2000	1705	This work
**OSARIP**	60:1	0.4 MPa	2000	4147	This work
**OSARIP**	20:1	0.4 MPa	5000	185	This work

**PIP-MWCNTs/PEI/PES**	21.4:1	0.4 MPa	2000	7	37
**DAPP/TMC**	20:1	0.3 MPa	2000	~3	38
**TMC-BPEI-EDTA**	24:1	1.0 MPa	2500	9	39
**PES/ PIP-PHF/ TMC**	21.4：1	0.6 MPa	2000	13	40
**PEI/ TMC**	20:1	0.8 MPa	2000	20	41
**CQDs-NH_2/TMC**	30:1	0.2 MPa	2000	14	42
**QEDTP/PEI/TMC**	120:1	0.6 MPa	2000	16	43
**CNC-COOH/PEI/TMC**	30:1	0.8 MPa	2000	12	44
**（MWCNTs-COOK）-PEI/TMC**	20:1	0.3 MPa	2000	58	45
**PA-g-AS-Fe**	20:1	0.6 MPa	2000	81	46
**（PES-GO）/PEI/TMC**	20:1	0.3 MPa	2000	16	47
**UIO-66-NH_2/PIP/TMC**	30.6:1	1 MPa	2000	79	48
**NF-IL-2.0%**	20:1	0.6 MPa	2100	8	49
**PEI-LDH/GA**	10:1	0.5 MPa	1000	19	50
**PDA-PEI-NF270**	30:1	-	12000	7	51
**PDA-PEI-DL**	30:1	-	12000	5	51
**PDA-PEI-DK**	30:1	-	12000	59	51
**PEI/GQDs-NH2/TMC**	20:1	0.3 MPa	2000	28	52
**MBCN-0.02**	73:1	0.4 MPa	2000	24	53
**PIP-TMC-PEI**	21.4:1	0.4 MPa	2000	33	26
**SPE-PEI600**	150:1	0.6 MPa	2000	12	54
**PSS/PAH (pH 2.7)**	60:1	0.4 MPa	2000	430	55
**PSS/PAH 2**	20:1	0.4 MPa	2000	176	55
**PSS/PAH**	60:1	0.4 MPa	2000	75	56
**SIP-PEI-TMC**	20:1	1.5 MPa	2000	15	57
**QBPD/PEI**	50:1	0.6 MPa	2000	5	58

Supplementary Table 8. Summary of Li^{+}/Mg^{2+} selectivity of MOFs membranes, COFs membrane, and POPs membranes reported in literatures.

Membrane	$S_{Mg/Li}$	Ref.
HKUST-1-PSS	1815	59
UiO-66-$(COOH)_2$	1590	60
UiO-66-COOH	200	61
UiO-66-NH_2	60	62
COF ($TpBDMe_2$)	217	63
COF-EB_1BD_1-PAN	443	64
COF-4EO-PAN	64	65
PIM-TB-$FeCl_3$	176	66
PIM-BzMA-TB	33	67
POCs-CC3	284	68
MXene-(Ti_3C_2Tx)-PSS	28	69

Note: We must emphasize here that the examining of ion separation performance of all these membranes is based on a slow diffusion process that relies on concentration gradients without applied pressure, rather than a pressure-driven process like nanofiltration membranes. Although these two processes are not comparable in terms of processing capacity and scale application (obviously pressure-driven nanofiltration membranes have more advantages), we only compare their separation selectivity without considering other aspects.

2.9 PA NF membranes prepared from different conditions

Supplementary Table 9. Different PA NF membranes prepared from OSARIP (M1-M8) and their corresponding preparation conditions. The reaction temperature is 35 ºC.

PA NF membrane	Conditions
**M1**	TMC: 2 g L^{-1}, PIP: 5 g L^{-1}, DDP: 0.1 g L^{-1}
**M2**	TMC: 2 g L^{-1}, PIP 5: g L^{-1}, DDP: 0.125 g L^{-1}
**M3**	TMC: 2 g L^{-1}, PIP: 5 g L^{-1}, DDP: 0.05 g L^{-1}
**M4**	TMC: 2 g L^{-1}, PIP: 5 g L^{-1}, DDP: 0.15 g L^{-1}
**M5**	TMC: 2 g L^{-1}, PIP: 2.5 g L^{-1}, DDP: 0.1 g L^{-1}
**M6**	TMC: 2 g L^{-1}, PIP: 5 g L^{-1}, DDP: 0.01 g L^{-1}
**M7**	TMC: 2 g L^{-1}, PIP: 5 g L^{-1}, DDP: 0 g L^{-1}
**M8**	TMC: 2 g L^{-1}, PIP: 2.5 g L^{-1}, DDP: 0 g L^{-1}

Supplementary Table 10. Salt rejection and water permeance of PA NF membranes (M1-M8). The concentration of feed solution is 1000 ppm. The applied pressure is 4 bar.

PA NF membrane	Rejection (%)						Water permeance
	Na_2SO_4	$MgSO_4$	$MgCl_2$	$CaCl_2$	NaCl	LiCl	($Lm^{-2}h^{-1}bar^{-1}$)
M1	99.73	99.91	99.29	99.33	40.32	43.00	8.5
M2	99.65	99.83	98.96	98.26	35.15	30.36	7.1
M3	99.23	99.56	98.46	97.20	27.87	25.31	7.1
M4	99.70	99.81	98.32	97.10	29.32	27.54	7.8
M5	98.47	98.83	95.47	94.19	23.86	24.71	9.2
M6	98.90	98.88	93.67	95.94	24.58	23.67	11.3
M7	98.13	97.79	91.21	88.42	19.41	20.37	14.1
M8	99.30	98.59	57.77	65.17	12.53	15.67	16.3

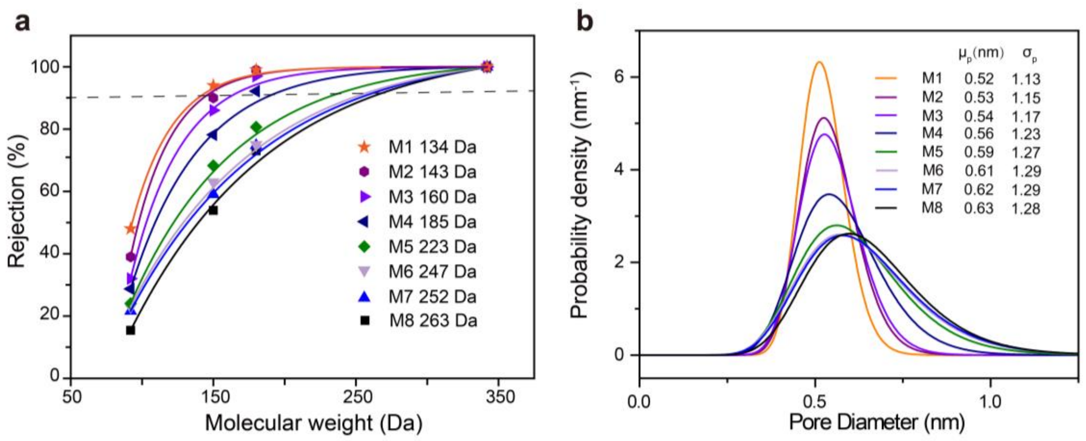

Supplementary Fig. 15. (a) Rejection of neutral solutes with different molecular weights by the PA NF membranes (M1-M8), and (b) corresponding pore size distribution derived from the rejection curve of neutral solutes.

2.10 PA NF membranes prepared from OSARIP by using PEI and TMC as the monomers

Supplementary Table 11. The conditions for preparing PA NF membranes via OSARIP using PEI and TMC as the reaction monomers. The molecular weight of PEI is 600.

	Preparation conditions
M1-PEI	TMC: 2 g L^{-1}, PEI: 10 gL^{-1}, DDP: 0.1 g L^{-1}
M2-PEI	TMC: 2 g L^{-1}, PEI: 10 g L^{-1}, DDP: 0.15 g L^{-1}
M3-PEI	TMC: 2 g L^{-1}, PEI: 10 g L^{-1}, DDP: 0.2 g L^{-1}
M4-PEI	TMC: 2 g L^{-1}, PEI: 10 g L^{-1}, DDP: 0.05 g L^{-1}
M5-PEI	TMC: 2 g L^{-1}, PEI: 10 g L^{-1}, DDP: 0 g L^{-1}

Supplementary Table 12. The rejection of Li^+ and Mg^{2+} and Li^+/Mg^{2+} selectivity of the PA NF membranes prepared from OSARIP by using PEI and TMC as monomers for treating binary salt mixture solutions as feed. The salt concentration of feed solution is 2000 ppm. The Mg^{2+}/Li^+ mass ratio of feed solution is 20:1.

	Li^+ rejection	Mg^{2+} rejection	$S_{Mg/Li}$
M1-PEI	52.23%	98.11%	25
M2-PEI	29.53%	93.96%	12
M3-PEI	25.15%	91.65%	9
M4-PEI	19.22%	88.53%	7
M5-PEI	8.21%	67.61%	3

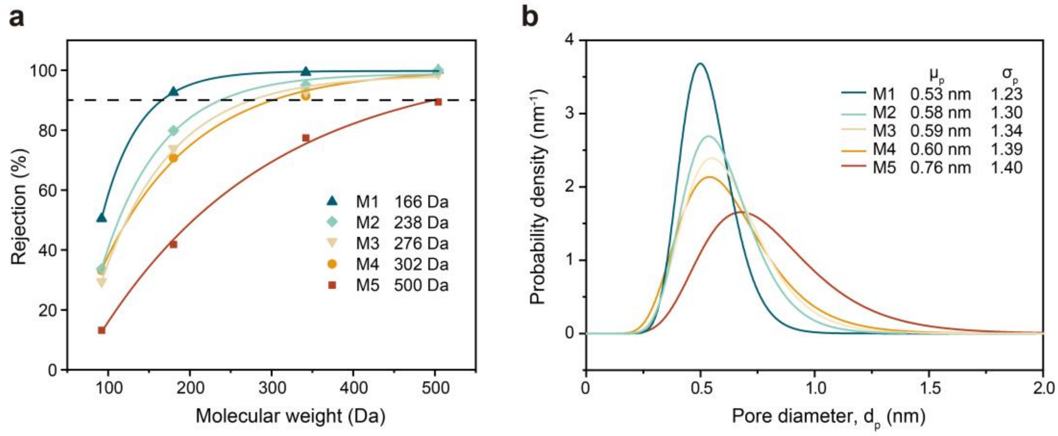

Supplementary Fig. 16. (a) The rejection of neutral solutes with different molecular weights and **(b)** the pore size distribution derived from the rejection curve of neutral solutes by the PA NF membrane prepared from OSARIP by using PEI and TMC as monomers.

Note: As presented in Supplementary Tables 11, 12 and Supplementary Fig. 16, the OSARIP process can also enhance the pore size uniformity of the PA NF membrane prepared from PEI and TMC. It is well known that PA NF membranes prepared from the IP reaction of PEI and TMC is strongly positive. When using these positive PA NF membranes for separating Li^+ and Mg^{2+} from the binary salt mixture solution, a higher Mg^{2+} rejection is presented by the membrane with narrower pore size distribution,

which is similar to the PA NF membranes prepared using PIP and TMC as monomers. But, a higher Li^+ rejection is also presented by the positive PA NF membranes and no negative rejection is obtained. Meanwhile, the Li^+ rejection increases with narrowing the pore size distribution. The change of Li^+ rejection by the PA NF membrane of PEI and TMC is contrast to that of the PA NF membranes of PIP and TMC. We postulate that this difference relies mainly on the naturally positive property of PEI-TMC-based NF membrane, which dramatically reduces the permeation of Li^+ via Donnan exclusion by hindering the Li^+ partition and electrostatic interaction for slowing down the transport of Cl^-. This is not beneficial for obtaining a high recovery of Li^+ when using these positively charged PA NF membranes for extracting Li^+ from brine. These findings also prove the superiority of negatively charged PA NF membranes with highly uniform pore size distribution for extracting Li^+ from brine.

3. Supplementary references

1. Tang, C. Y., Kwon, Y.-N. & Leckie, J. O. Effect of membrane chemistry and coating layer on physiochemical properties of thin film composite polyamide RO and NF membranes: I. FTIR and XPS characterization of polyamide and coating layer chemistry. *Desalination* **242**, 149–167 (2009).
2. Karan, S., Jiang, Z. & Livingston, A. G. Sub–10 nm polyamide nanofilms with ultrafast solvent transport for molecular separation. *Science* **348**, 1347–1351 (2015).
3. Yaroshchuk, A. & Bruening, M. L. An analytical solution of the solution-diffusion-electromigration equations reproduces trends in ion rejections during nanofiltration of mixed electrolytes. *J. Membr. Sci.* **523**, 361–372 (2017).
4. Yaroshchuk, A., Bruening, M. L. & Licón Bernal, E. E. Solution-Diffusion–Electro-Migration model and its uses for analysis of nanofiltration, pressure-retarded osmosis and forward osmosis in multi-ionic solutions. *J. Membr. Sci.* **447**, 463–476 (2013).
5. Wang, R., He, R., He, T., Elimelech, M. & Lin, S. Performance metrics for nanofiltration-based selective separation for resource extraction and recovery. *Nat. Water* **1**, 291–300 (2023).

6. Zhu, C.-Y. *et al.* Polyamide nanofilms with linearly-tunable thickness for high performance nanofiltration. *J. Membr. Sci.* **627**, 119142 (2021).
7. Nightingale, E. R. Jr. Phenomenological Theory of Ion Solvation. Effective Radii of Hydrated Ions. *J. Phys. Chem.* **63**, 1381–1387 (1959).
8. Marcus, Y. Thermodynamics of solvation of ions. Part 6.—The standard partial molar volumes of aqueous ions at 298.15 K. *J. Chem. Soc., Faraday Trans.* **89**, 713–718 (1993).
9. Zhang, R. *et al.* Polyamide Nanofiltration Membranes from Surfactant-Assembly Regulated Interfacial Polymerization: The Effect of Alkyl Chain. *Macromol. Chem. Phys.* **222**, 2100222 (2021).
10. Liang, Y. *et al.* Polyamide nanofiltration membrane with highly uniform sub-nanometre pores for sub-1 Å precision separation. *Nat. Commun.* **11**, 2015 (2020).
11. Yuan, B. *et al.* Ultrathin Polyamide Membrane with Decreased Porosity Designed for Outstanding Water-Softening Performance and Superior Antifouling Properties. *ACS Appl. Mater. Interfaces* **10**, 43057–43067 (2018).
12. Tian, B. *et al.* Nanofiltration membrane combining environmental-friendly polycarboxylic interlayer prepared from catechol for enhanced desalination performance. *Desalination* **512**, 115118 (2021).
13. Gong, G., Wang, P., Zhou, Z. & Hu, Y. New Insights into the Role of an Interlayer for the Fabrication of Highly Selective and Permeable Thin-Film Composite Nanofiltration Membrane. *ACS Appl. Mater. Interfaces* **11**, 7349–7356 (2019).
14. Shen, K., Li, P., Zhang, T. & Wang, X. Salt-tuned fabrication of novel polyamide composite nanofiltration membranes with three-dimensional turing structures for effective desalination. *J. Membr. Sci.* **607**, 118153 (2020).
15. Hao, Y. *et al.* An ultrahighly permeable-selective nanofiltration membrane mediated by an in situ formed interlayer. *J. Mater. Chem. A* **8**, 5275–5283 (2020).
16. Gao, S. *et al.* Ultrathin Polyamide Nanofiltration Membrane Fabricated on Brush-Painted Single-Walled Carbon Nanotube Network Support for Ion Sieving. *ACS Nano* **13**, 5278–5290 (2019).
17. Huang, S. *et al.* Polyamide Nanofiltration Membranes Incorporated with Cellulose

Nanocrystals for Enhanced Water Flux and Chlorine Resistance. *ACS Sustainable Chem. Eng.* **7**, 12315–12322 (2019).

18. Wang, Q. *et al.* Designing High-Performance Nanofiltration Membranes for High-Salinity Separation of Sulfate and Chloride in the Chlor-Alkali Process. *Ind. Eng. Chem. Res.* **58**, 12280–12290 (2019).
19. Du, Y., Qiu, W.-Z., Lv, Y., Wu, J. & Xu, Z.-K. Nanofiltration Membranes with Narrow Pore Size Distribution via Contra-Diffusion-Induced Mussel-Inspired Chemistry. *ACS Appl. Mater. Interfaces* **8**, 29696–29704 (2016).
20. Yuan, B. *et al.* Semi-aromatic polyamide nanofiltration membranes with tuned surface charge and pore size distribution designed for the efficient removal of Ca2+ and Mg2+. *Sep. Purif. Technol.* **220**, 162–175 (2019).
21. Liu, Y. *et al.* A Facile and Scalable Fabrication Procedure for Thin-Film Composite Membranes: Integration of Phase Inversion and Interfacial Polymerization. *Environ. Sci. Technol.* **54**, 1946–1954 (2020).
22. Cheng, X. *et al.* Toward Enhancing Desalination and Heavy Metal Removal of TFC Nanofiltration Membranes: A Cost-Effective Interface Temperature-Regulated Interfacial Polymerization. *ACS Appl. Mater. Interfaces* **13**, 57998–58010 (2021).
23. Zhan, Z.-M., Xu, Z.-L., Zhu, K.-K. & Tang, Y.-J. How to understand the effects of heat curing conditions on the morphology and performance of polypiperazine-amide NF membrane. *J. Membr. Sci.* **597**, 117640 (2020).
24. Lu, Y. *et al.* Two-dimensional fractal nanocrystals templating for substantial performance enhancement of polyamide nanofiltration membrane. *Proceedings of the National Academy of Sciences* **118**, e2019891118 (2021).
25. Zhan, Z.-M. *et al.* Superior nanofiltration membranes with gradient cross-linked selective layer fabricated via controlled hydrolysis. *J. Membr. Sci.* **604**, 118067 (2020).
26. Yang, Z. *et al.* Dual-skin layer nanofiltration membranes for highly selective Li+/Mg2+ separation. *J. Membr. Sci.* **620**, 118862 (2021).
27. Peng, H. *et al.* Phosphonium Modification Leads to Ultrapermeable Antibacterial Polyamide Composite Membranes with Unreduced Thickness. *Adv. Mater.* **32**,

2001383 (2020).

28. Huang, B.-Q., Tang, Y.-J., Zeng, Z.-X. & Xu, Z.-L. Microwave heating assistant preparation of high permselectivity polypiperazine-amide nanofiltration membrane during the interfacial polymerization process with low monomer concentration. *J. Membr. Sci.* **596**, 117718 (2020).
29. Liu, Y., Wang, X., Yang, H., Xie, Y. F. & Huang, X. Preparation of nanofiltration membranes for high rejection of organic micropollutants and low rejection of divalent cations. *J. Membr. Sci.* **572**, 152–160 (2019).
30. Huang, B.-Q., Xu, Z.-L., Ding, H., Miao, M.-C. & Tang, Y.-J. Antifouling sulfonated polyamide nanofiltration hollow fiber membrane prepared with mixed diamine monomers of BDSA and PIP. *RSC Adv.* **7**, 56629–56637 (2017).
31. Dolar, D., Vuković, A., Ašperger, D. & Košutić, K. Effect of water matrices on removal of veterinary pharmaceuticals by nanofiltration and reverse osmosis membranes. *J. Environ. Sci.* **23**, 1299–1307 (2011).
32. Qin, Y. *et al.* Performance enhancement of nanofiltration membranes via surface modification with a novel acylation reagent. *J. Appl. Polym. Sci.* **138**, 50315 (2021).
33. Schaep, J., Van der Bruggen, B., Vandecasteele, C. & Wilms, D. Influence of ion size and charge in nanofiltration. *Sep. Purif. Technol.* **14**, 155–162 (1998).
34. Van der Bruggen, B., Koninckx, A. & Vandecasteele, C. Separation of monovalent and divalent ions from aqueous solution by electrodialysis and nanofiltration. *Water Res.***38**, 1347–1353 (2004).
35. Yang, Z. *et al.* Tannic Acid/Fe3+ Nanoscaffold for Interfacial Polymerization: Toward Enhanced Nanofiltration Performance. *Environ. Sci. Technol.* **52**, 9341–9349 (2018).
36. Ren, T. *et al.* Influence of inorganic salt on retention of ibuprofen by nanofiltration. *Sep. Purif. Technol.* **189**, 382–388 (2017).
37. Zhang, H.-Z., Xu, Z.-L., Ding, H. & Tang, Y.-J. Positively charged capillary nanofiltration membrane with high rejection for Mg2+ and Ca2+ and good separation for Mg^{2+} and Li^{+}. *Desalination* **420**, 158–166 (2017).
38. Li, X. *et al.* Preparation and characterization of positively charged polyamide

composite nanofiltration hollow fiber membrane for lithium and magnesium separation. *Desalination* **369**, 26–36 (2015).

39. Li, W. *et al.* A positively charged composite nanofiltration membrane modified by EDTA for LiCl/MgCl2 separation. *Sep. Purif. Technol.* **186**, 233–242 (2017).

40. Shen, Q., Xu, S.-J., Xu, Z.-L., Zhang, H.-Z. & Dong, Z.-Q. Novel thin-film nanocomposite membrane with water-soluble polyhydroxylated fullerene for the separation of Mg^{2+}/Li^{+} aqueous solution. *J. Appl. Polym. Sci.* **136**, 48029 (2019).

41. Xu, P. *et al.* Positive charged PEI-TMC composite nanofiltration membrane for separation of Li^{+} and Mg^{2+} from brine with high Mg^{2+}/Li^{+} ratio. *Desalination* **449**, 57–68 (2019).

42. Guo, C. *et al.* Amino-rich carbon quantum dots ultrathin nanofiltration membranes by double "one-step" methods: Breaking through trade-off among separation, permeation and stability. *Chem. Eng. J.* **404**, 127144 (2021).

43. Xu, Y. *et al.* High performance Mg^{2+}/Li^{+} separation membranes modified by a bis-quaternary ammonium salt. *Desalination* **526**, 115519 (2022).

44. Guo, C. *et al.* Ultra-thin double Janus nanofiltration membrane for separation of Li^{+} and Mg^{2+}: "Drag" effect from carboxyl-containing negative interlayer. *Sep. Purif. Technol.* **230**, 115567 (2020).

45. Xu, P., Hong, J., Xu, Z., Xia, H. & Ni, Q.-Q. MWCNTs-COOK-assisted high positively charged composite membrane: Accelerating Li^{+} enrichment and Mg^{2+} removal. *Compos. Part B: Eng.* **212**, 108686 (2021).

46. Liu, Y. *et al.* A nanofiltration membrane with positively and negatively charged groups by grafted p-aminosalicylic acid-Fe(III) chelation for Li^{+}/Mg^{2+} efficient separation. *Sep. Purif. Technol.* **308**, 122968 (2023).

47. Xu, P. *et al.* "Bridge" graphene oxide modified positive charged nanofiltration thin membrane with high efficiency for Mg^{2+}/Li^{+} separation. *Desalination* **488**, 114522 (2020).

48. Yuan, B. *et al.* Polyamide nanofiltration membrane fine-tuned via mixed matrix ultrafiltration support to maximize the sieving selectivity of Li^{+}/Mg^{2+} and Cl^{-}/SO_4^{2-}. *Desalination* **538**, 115929 (2022).

49. Wu, H. *et al.* A novel nanofiltration membrane with [MimAP][Tf2N] ionic liquid for utilization of lithium from brines with high Mg^{2+}/Li^{+} ratio. *J. Membr. Sci.* **603**, 117997 (2020).

50. Ni, H., Wang, N., Yang, Y., Shen, M. & An, Q.-F. Positively-charged nanofiltration membrane constructed by polyethyleneimine/layered double hydroxide for Mg^{2+}/Li^{+} separation. *Desalination* **548**, 116256 (2023).

51. Ashraf, M. A. *et al.* Enhancement in Li^{+}/Mg^{2+} separation from salt lake brine with PDA–PEI composite nanofiltration membrane. *J. Appl. Polym. Sci.* **137**, 49549 (2020).

52. Xu, P., Hong, J., Xu, Z., Xia, H. & Ni, Q.-Q. Novel aminated graphene quantum dots (GQDs-NH2)-engineered nanofiltration membrane with high Mg^{2+}/Li^{+} separation efficiency. *Sep. Purif. Technol.* **258**, 118042 (2021).

53. Bi, Q., Zhang, C., Liu, J., Liu, X. & Xu, S. Positively charged zwitterion-carbon nitride functionalized nanofiltration membranes with excellent separation performance of Mg^{2+}/Li^{+} and good antifouling properties. *Sep. Purif. Technol.* **257**, 117959 (2021).

54. Lu, D. *et al.* Constructing a selective blocked-nanolayer on nanofiltration membrane via surface-charge inversion for promoting Li^{+} permselectivity over Mg2+. *J. Membr. Sci.* **635**, 119504 (2021).

55. He, R. *et al.* Polyelectrolyte-based nanofiltration membranes with exceptional performance in Mg2+/Li+ separation in a wide range of solution conditions. *J. Membr. Sci.* **663**, 121027 (2022).

56. He, R. *et al.* Unprecedented Mg^{2+}/Li^{+} separation using layer-by-layer based nanofiltration hollow fiber membranes. *Desalination* **525**, 115492 (2022).

57. Li, Y. *et al.* Polyamide nanofiltration membranes with rigid–flexible microstructures for high-efficiency Mg^{2+}/Li^{+} separation. *Sep. Purif. Technol.* **306**, 122552 (2023).

58. Feng, Y., Peng, H. & Zhao, Q. Fabrication of high performance Mg^{2+}/Li^{+} nanofiltration membranes by surface grafting of quaternized bipyridine. *Sep. Purif. Technol.* **280**, 119848 (2022).

59. Guo, Y., Ying, Y., Mao, Y., Peng, X. & Chen, B. Polystyrene Sulfonate Threaded through a Metal–Organic Framework Membrane for Fast and Selective Lithium-Ion Separation. *Angew. Chem. Int. Ed.* **55**, 15120–15124 (2016).
60. Lu, J. *et al.* Efficient metal ion sieving in rectifying subnanochannels enabled by metal–organic frameworks. *Nat. Mater.* **19**, 767–774 (2020).
61. Lu, J. *et al.* Ultraselective Monovalent Metal Ion Conduction in a Three-Dimensional Sub-1 nm Nanofluidic Device Constructed by Metal–Organic Frameworks. *ACS Nano* **15**, 1240–1249 (2021).
62. Xu, T. *et al.* Highly Cation Permselective Metal–Organic Framework Membranes with Leaf-Like Morphology. *ChemSusChem* **12**, 2593–2597 (2019).
63. Sheng, F. *et al.* Efficient Ion Sieving in Covalent Organic Framework Membranes with Sub-2-Nanometer Channels. *Adv. Mater.* **33**, 2104404 (2021).
64. Hou, L. *et al.* Understanding the Ion Transport Behavior across Nanofluidic Membranes in Response to the Charge Variations. *Adv. Funct. Mater.* **31**, 2009970 (2021).
65. Yang, J., Li, L. & Tang, Z. An efficient lithium extraction pathway in covalent organic framework membranes. *Matter* **4**, 2666–2668 (2021).
66. Zhang, C., Mu, Y., Zhao, S., Zhang, W. & Wang, Y. Lithium extraction from synthetic brine with high Mg^{2+}/Li^{+} ratio using the polymer inclusion membrane. *Desalination* **496**, 114710 (2020).
67. Tan, R. *et al.* Hydrophilic microporous membranes for selective ion separation and flow-battery energy storage. *Nat. Mater.* **19**, 195–202 (2020).
68. Xu, T. *et al.* Highly Ion-Permselective Porous Organic Cage Membranes with Hierarchical Channels. *J. Am. Chem. Soc.* **144**, 10220–10229 (2022).
69. Lu, Z., Wu, Y., Ding, L., Wei, Y. & Wang, H. A Lamellar MXene ($Ti_3C_2T_x$)/PSS Composite Membrane for Fast and Selective Lithium-Ion Separation. *Angew. Chem. Int. Ed.* **60**, 22265–22269 (2021).
